# Paternal Broader Autism Phenotype and Adolescent Avoidant Attachment as Independent Predictors of Negative Symptoms in Early Psychosis

**DOI:** 10.1002/brb3.71579

**Published:** 2026-07-09

**Authors:** Gokcen Ilcioglu Ekici, Ceren Sena Akyuz Coskuner, Merve Onat, Esra Cop

**Affiliations:** ^1^ Department of Child and Adolescent Psychiatry, Ankara Bilkent City Hospital Affiliated With Ankara Yildirim Beyazit University Faculty of Medicine Ankara Türkiye; ^2^ Department of Child and Adolescent Psychiatry Ankara Bilkent City Hospital Ankara Türkiye

**Keywords:** adolescent psychosis, avoidant attachment, negative symptoms, parental broader autism phenotype

## Abstract

**Introduction:**

Familial neurodevelopmental traits and early relational experiences are increasingly recognized as contributors to heterogeneity in psychosis outcomes. However, little is known about how parental broader autism phenotype (BAP) and adolescent attachment jointly relate to symptom severity and treatment adherence in early‐onset psychosis.

**Methods:**

This cross‐sectional study enrolled 42 adolescents (aged 12–17 years; 88.1% schizophrenia, 11.9% schizoaffective disorder) and their biological parents. Parental BAP was assessed using the Autism Spectrum Quotient‐Revised (AQ‐R), adolescent attachment with the Psychosis Attachment Measure (PAM), symptom severity with the Scale for the Assessment of Positive/Negative Symptoms (SAPS/SANS), and parent‐reported medication adherence with the MARS‐5. Hierarchical multiple regression analyses were conducted to test the hypotheses.

**Results:**

Parental BAP did not predict adolescent attachment. However, the model predicting negative symptoms was significant (*R* ^2^ = 0.439, *p* = 0.022). Higher paternal AQ‐R scores (*β* = 0.45, *p* = 0.016) and avoidant attachment (*β* = 0.46, *p* = 0.005) independently predicted more severe negative symptoms. Medication adherence was not associated with parental BAP; instead, longer DUP was the only robust correlate of poorer adherence (*β* = −0.56, *p* = 0.005). Sensitivity analyses using AQ‐50 scores yielded consistent findings.

**Conclusion:**

Paternal autistic traits and adolescent avoidant attachment may represent independent developmental and relational factors associated with negative symptoms in early psychosis. These findings underscore the need for family‐informed assessment and intervention strategies, particularly targeting paternal characteristics and attachment dynamics. Given the modest sample size and cross‐sectional design, replication in larger longitudinal cohorts is warranted.

## Graphical abstract

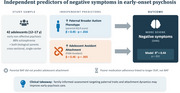

## 1 Introduction

Schizophrenia is a well‐recognized psychiatric disorder, yet its precise etiology is not fully understood. Current evidence suggests that schizophrenia emerges from the complex interplay of genetic, environmental, neurodevelopmental, and biochemical factors (Farsi and Sheng 2023; Owen et al. 2016). In adolescents, this interaction may be particularly important because psychosis develops during a period in which identity formation, social cognition, family relationships, and treatment engagement are still evolving (Patel et al., 2021). Stressful life events and traumatic experiences during critical developmental periods have been associated with increased vulnerability to psychosis onset and poorer clinical course (Rosenthal et al., 2020).

Attachment theory provides one framework for understanding how early relational experiences may shape vulnerability and adaptation in psychosis. Early caregiver–infant relationships contribute to the development of internal working models that guide emotion regulation and social functioning (Johnson et al. 2007; Owens et al. 1995). Secure attachment fosters lifelong psychological resilience (Darling Rasmussen et al. 2019), whereas insecure attachment increases risk for negative mental health outcomes (Bakermans‐Kranenburg and Van Ijzendoorn 2009), including the risk of developing psychosis (Herstell et al. 2021; Mathews et al. 2016). Consistent with this literature, several studies have reported a higher frequency of insecure attachment among individuals with psychosis compared with the general population (Carr et al. 2018). In psychosis, insecure attachment appears to be more consistently associated with poorer social functioning, social withdrawal (De With et al., 2023), interpersonal threat sensitivity, and difficulties in help‐seeking (Sood et al., 2022), whereas its association with overall symptom severity is generally modest (Carr et al. 2018). Therefore, attachment style may be particularly relevant in adolescent psychosis, where family relationships remain central to daily functioning, clinical monitoring, and adherence to care.

The broader autism phenotype (BAP) encompasses subthreshold social, cognitive, and behavioral characteristics that resemble those observed in autism spectrum disorder but are more subtle (Dawson et al. 2002). Empirical studies have shown that BAP traits cluster more frequently in families of individuals with ASD, indicating heritability (Losh and Piven 2007; Rubenstein et al. 2019). Importantly, these subclinical autistic traits are not confined to ASD; they have also been reported among other psychiatric disorders, with psychosis being one of them (Kincaid et al. 2017). Moreover, the phenotypic overlap between psychosis and ASD—encompassing impairments in social communication, restricted behaviors, and social cognition deficits—points toward potential commonalities in their underlying neurodevelopmental pathways (Ruas Resende et al. 2024). Although direct evidence of elevated BAP in parents of adolescents with psychosis remains limited, the heritability of autistic traits implies that these parents may exhibit more pronounced BAP characteristics compared to the general population.

Parental psychological characteristics shape parenting behaviors and influence children's attachment patterns (Rabadán Rubio et al. 2019; Vafaeenejad et al. 2019). Sensitive and supportive parenting tends to promote security, while inconsistent, distant, or authoritarian styles often lead to insecurity (Wang 2023). In this context, parental BAP may be relevant because subclinical autistic traits can involve subtle differences in social reciprocity, emotional expressiveness, and cognitive flexibility. Such characteristics do not necessarily imply impaired parenting, but they may influence the quality of emotional attunement and communication within the parent–child relationship. Given that secure attachment is frequently disrupted in individuals with psychosis (Carr et al. 2018), it is plausible to hypothesize that parental BAP may contribute to attachment‐related vulnerability by influencing relational processes within the parent–child dyad.

In treating children and adolescents with severe psychiatric conditions, such as psychosis, parents play a critical role by monitoring symptom progression, ensuring adherence to interventions, and providing essential emotional support. Research has consistently shown that close parental involvement in the therapeutic process significantly improves clinical outcomes in pediatric psychiatric populations (MacPherson et al. 2021). Conversely, poor treatment adherence in psychosis has been linked to worse functional outcomes and a diminished quality of life (Verdoux et al. 2021). However, both insecure attachment and parental BAP have been independently linked to challenges in treatment engagement (McGonagle et al. 2021; Parr et al. 2015). The combined influence of these familial factors on clinical outcomes in adolescent psychosis remains a significant gap in the literature.

In light of these observations, the current study specifically examines two primary questions. First, is there an association between attachment patterns in adolescents diagnosed with non‐affective psychotic disorders and the presence of BAP characteristics in their parents? Second, to what extent are these interrelated factors—parental BAP and adolescent attachment—associated with symptom severity and parent‐reported treatment adherence? We hypothesized that higher parental BAP would be associated with greater adolescent attachment insecurity, and that both parental BAP and insecure attachment would be associated with more severe symptoms and poorer treatment adherence. By clarifying these dynamics, we aim to advance the understanding of how familial attachment relationships and subclinical autistic traits can inform more tailored interventions for adolescents with non‐affective psychotic disorders.

## 2 Materials and Methods

### 2.1 Study Design and Participants

This single‐center, cross‐sectional study was conducted at the Ankara Bilkent City Hospital with ethics approval obtained from the institutional review board (No: E2‐23‐4770). Written informed consent from legal guardians and assent from adolescents were obtained. The study adhered to the Declaration of Helsinki and STROBE guidelines.

Adolescents aged 12–17 years with DSM‐5 non‐affective psychotic disorders were eligible. The final sample comprised 42 patients (schizophrenia: *n* = 37, 88.1%; schizoaffective disorder: *n* = 5, 11.9%) recruited between October 2023 and May 2024. Inclusion criteria required co‐residence with both biological parents, clinician‐judged age‐appropriate cognitive functioning, and adequate language skills. Exclusion criteria were other psychotic disorders (e.g., brief psychotic disorder and schizophreniform disorder), primary mood or substance use disorders, intellectual disability, or severe parental illness precluding participation. Intellectual disability was operationally defined as a Full‐Scale IQ < 70 based on previously administered Wechsler Intelligence Scale for Children–Revised (WISC‐R) scores documented in medical records.

Although no a priori power analysis was conducted due to the exploratory nature of this study, the sample size is comparable to or larger than those of previous investigations examining BAP and attachment‐related processes in clinical populations. Therefore, the present findings should be interpreted as hypothesis‐generating and in need of replication in larger samples.

### 2.2 Procedure and Measures

Participants and parents were recruited from inpatient and outpatient units. Data collection included clinician‐rated interviews and self/parent‐report questionnaires. For each adolescent, both biological parents completed the parent‐report questionnaires independently. Psychotic symptoms (SAPS/SANS) were rated by a single psychiatrist blinded to other data.

*Sociodemographic* and *clinical data* were collected via a structured caregiver form and cross‐validated with medical records. Duration of Untreated Psychosis (DUP) was operationalized as the interval from the onset of the first continuous psychotic symptom to the initiation of adequate antipsychotic treatment. Whenever possible, DUP estimates derived from caregiver interviews were cross‐validated against electronic medical records to reduce recall bias.

*Parental autistic traits* were assessed using the Autism Spectrum Quotient (AQ; Baron‐Cohen et al. 2001). Consistent with psychometric recommendations (Stevenson and Hart 2017), we used a Likert‐based scoring method. Our primary analyses utilized the 37‐item revised Turkish version (AQ‐R), which has demonstrated good psychometric properties (Aykan and Kalaycioğlu 2020; Cronbach's *α* = 0.789). The standard 50‐item version (AQ‐50) was used only for sensitivity analyses.

*Attachment* was evaluated using the Psychosis Attachment Measure (PAM; Berry et al. 2006), a self‐report instrument designed for assessing attachment‐related thoughts, feelings, and behaviors in people with psychosis. The PAM includes two dimensions: PAM‐Anxious, reflecting fears of rejection or abandonment and a heightened need for reassurance, and PAM‐Avoidant, reflecting discomfort with closeness, emotional distance, and reluctance to rely on others. Higher scores indicate greater attachment insecurity in the corresponding dimension. The Turkish version has demonstrated acceptable‐to‐excellent internal consistency, with Cronbach's *α* values ranging from 0.74 to 0.92 (Şehit Burhan et al. 2022).

*Medication adherence* was measured using the 5‐item MARS‐5, completed by parents as a parent‐proxy report (Thompson et al. 2000). The Turkish version has shown acceptable reliability (Cronbach's *α* = 0.78; Sertel Berk et al. 2019).

*Psychotic symptom severity* was assessed using the Scale for the Assessment of Positive/Negative Symptoms (SAPS/SANS; Andreasen 1983, 1984). Turkish versions showed strong psychometrics; SAPS *α* = 0.844, SANS high inter‐rater and test–retest reliability (Erkoç et al. 1991a, 1991b).

### 2.3 Participant Flow

A total of 104 adolescents were screened at clinical triage for possible psychosis. Thirteen were excluded prior to full assessment because subsequent clinical evaluation did not support a DSM‐5 psychotic disorder. Ninety‐one adolescents proceeded to full assessment; 49 were excluded (parental refusal = 8, previously documented WISC‐R Full‐Scale IQ < 70 = 7, mood disorder = 23, other psychotic disorders = 11). Thus, the final study sample included 42 adolescents with non‐affective psychotic disorders. Parental data were obtained from both biological parents for each adolescent, comprising 42 mothers and 42 fathers.

### 2.4 Statistical Analysis

All analyses were conducted using SPSS (Version 24) with a significance level of *α* = 0.05. After calculating descriptive statistics, sex differences were assessed with independent *t*‐tests and bivariate relationships with Pearson's correlations.

Primary hypotheses were tested using hierarchical multiple regression analyses. All models controlled for adolescent age, sex, and DUP. The first set of models predicted attachment scores from parental AQ‐R scores. The second set predicted SANS scores from parental AQ‐R and adolescent attachment scores, and MARS‐5 scores from parental AQ‐R alone. Multicollinearity was assessed using variance inflation factor (VIF) values. Sensitivity analyses were performed by rerunning the significant SANS model with AQ‐50 scores and by comparing parental AQ‐50 scores to normative data using one‐sample *t*‐tests.

Given the modest sample size relative to the number of predictors, the regression models may be at some risk of overfitting despite acceptable multicollinearity indices. Therefore, the findings should be regarded as preliminary and interpreted with caution. In addition, no formal correction for multiple comparisons was applied, as the study was primarily hypothesis‐generating. To mitigate Type I error concerns, interpretation focused on convergence across effect sizes and consistency between primary and sensitivity analyses.

## 3 Results

### 3.1 Sample Characteristics

The sociodemographic and clinical characteristics of the 42 adolescents and their parents are detailed in Table 1. The sample consisted of an equal number of male and female adolescents (*n* = 21, 50.0%) with a mean age of 15.67 years (SD = 1.30). The majority of participants were receiving treatment in an inpatient setting (*n* = 38, 90.5%). The mean DUP was 7.95 weeks (SD = 7.90).

**TABLE 1:** Sample characteristics.

Variable	*n* (%) or mean ± SD	Range
Sociodemographic		
Age (years)	15.67 ± 1.3	12–17
Gender (male)	21 (50.0%)	
Service setting (inpatient)	38 (90.5%)	
Maternal age (years)	45.12 ± 5.8	30–58
Paternal age (years)	49.50 ± 6.2	33–60
Mother's education (years)	10.71 ± 4.5	5–16
Father's education (years)	12.50 ± 3.6	5–16
Family structure (nuclear)	36 (85.7%)	
Monthly family income ($)	1259.67 ± 930.6	294–4720
Family history of psychiatric disorders	20 (47.6%)	
Birth and neonatal history		
Maternal age at delivery (years)	28.95 ± 5.8	19–40
Gestational age at birth (weeks)	37.45 ± 1.7	34–42
Perinatal complications	16 (38.1%)	
NICU admission (any)	12 (%28.6)	
Clinical variables		
DUP (weeks)	7.95 ± 7.9	0–24
SANS total	36.39 ± 18.2	8–87
SAPS total	26.15 ± 13.9	7–56
PAM—anxiety	1.19 ± 0.85	0–3
PAM—avoidance	1.35 ± 0.66	0–2.8
MARS‐5	22.62 ± 3.6	10–25
Maternal AQ‐R total score	84.51 ± 15.1	57–127
Paternal AQ‐R total score	86.05 ± 13.6	62–110
Maternal AQ‐50 total score	114.26 ± 16.5	88–169
Paternal AQ‐50 total score	115.67 ± 14.8	92–151

*Note: N* = 42. Values are presented as *n* (%) for categorical variables and mean ± SD for continuous variables.

Abbreviations: DUP, duration of untreated psychosis; NICU, neonatal intensive care unit; SD, standard deviation.

### 3.2 Sex Differences

An independent samples *t*‐test was performed to assess sex differences across all primary continuous variables. A significant difference was found only for anxious attachment, with female adolescents reporting higher scores on the PAM‐Anxious (*M* = 1.47, SD = 0.88) compared to male adolescents (*M* = 0.92, SD = 0.75), *t*(40) = 2.156, *p* = 0.037. No other significant sex differences were observed.

### 3.3 Bivariate Correlational Analyses

Pearson correlation coefficients for the primary study variables are presented in Table 2. Several correlations relevant to the main hypotheses were statistically significant. A longer DUP was associated with poorer medication adherence (*r* = −0.36, *p* = 0.022) and higher paternal AQ‐R scores (*r* = 0.40, *p* = 0.014). Notably, higher paternal AQ‐R scores were also positively correlated with greater negative symptom severity (SANS total, *r* = 0.41, *p* = 0.010). Similarly, higher PAM‐Avoidant was strongly associated with more severe negative symptoms (SANS total, *r* = 0.43, *p* = 0.005).

**TABLE 2:** Descriptive statistics and bivariate correlations among key study variables.

Variable	*M* ± SD	1	2	3	4	5	6	7	8
1.Adolescent Age	15.67±1.30	—							
2. DUP (weeks)	7.95±7.90	.17	—						
3. Mother's AQ‐R	84.51±15.10	‐.03	.13	—					
4. Father's AQ‐R	86.05±13.60	.18	.40^*^	.31	—				
5. PAM‐Anxious	1.19±.85	‐.11	‐.03	‐.10	‐.01	—			
6. PAM‐Avoidant	1.35±.66	‐.19	‐.07	‐.26	‐.07	.19	—		
7. SAPS Total	26.15±13.90	.04	‐.06	.00	.08	.01	.03	—	
8. SANS Total	36.39±18.20	.05	.08	‐.10	.41^**^	.13	.43^**^	.43^**^	—
9. MARS‐5 Total	22.62±3.60	.02	‐.36^*^	.00	‐.10	‐.11	.24	.18	‐.05

*Note: N* = 42.

Abbreviations: AQ‐R, Autism Spectrum Quotient (37‐item revised total score); DUP, duration of untreated psychosis (in weeks); MARS‐5, Medication Adherence Report Scale (5‐item total score); PAM, Psychosis Attachment Measure (total scores); SANS, Scale for the Assessment of Negative Symptoms (total score); SAPS, Scale for the Assessment of Positive Symptoms (total score).

^*^ *p* < 0.05.

^**^ *p* < 0.01.

### 3.4 Exploratory Correlational Analyses

Exploratory correlational analyses revealed several other significant associations not specified in the primary hypotheses. Older maternal (*r* = −0.36, *p* = 0.019) and paternal age (*r* = −0.32, *p* = 0.037) were both related to poorer medication adherence. Regarding BAP subscales, higher scores on the paternal AQ‐Communication subscale were associated with greater negative symptoms (*r* = 0.44, *p* = 0.007). However, surprisingly, lower scores on the maternal AQ‐Communication subscale (reflecting better communication skills) were negatively correlated with higher PAM‐Avoidant attachment (*r* = −0.41, *p* = 0.009).

### 3.5 Primary Hypothesis Testing: Hierarchical Regression Models

To test our primary hypotheses, we conducted a series of hierarchical multiple regression analyses. The results of these analyses are summarized in Table 3. Collinearity diagnostics confirmed that multicollinearity was not a concern (all VIF < 10).

**TABLE 3:** Summary of Hierarchical Regression Analyses Predicting Adolescent Attachment and Clinical Outcomes.

	Model 1	Model 2	Model 3	Model 4
Predictor	PAM‐Anxious	PAM‐Avoidant	SANS Total	MARS‐5 Total
	β	β	β	β
Step 1: Covariates				
Adolescent Age	‐.09	‐.20	.15	.02
Gender (Female = 1)	‐.24	.00	‐.10	‐.18
DUP (weeks)	.00	‐.13	.00	‐.56^*^
*R* ^2^ for Step 1	.05	.05	.05	.26^*^
Step 2: Parental BAP				
Mother's AQ‐R	‐.10	‐.18	‐.14	‐.05
Father's AQ‐R	.03	.11	.45^*^	.12
Δ*R* ^2^ for Step 2	.01	.03	.19	.01
Step 3: Adolescent Attachment				
PAM‐Anxious	—	—	.00	—
PAM‐Avoidant	—	—	.46^*^	—
Δ*R* ^2^ for Step 3	—	—	.20^*^	—
Total *R* ^2^	.06	.08	.44^*^	.27
Adjusted *R* ^2^	‐.11	‐.09	.29	.14
*F* for Final Model	0.36	0.47	2.91^*^	2.09
*p* for Final Model	.873	.795	.022	.097

*Note*. β = Standardized beta coefficient from the final model. Δ*R* ^2^ = Change in *R*‐squared.

^*^ *p* < .05.

#### 3.5.1 Hypothesis 1: Predicting Adolescent Attachment Styles

Our first hypothesis, which posited that parental BAP traits would predict adolescent attachment styles, was not supported. After controlling for covariates, the addition of parental AQ‐R scores to the regression models did not explain a significant amount of additional variance in either PAM‐Anxious (Δ*R* ^2^ = 0.008, *p* = 0.868) or PAM‐Avoidant (Δ*R* ^2^ = 0.031, *p* = 0.618) attachment.

#### 3.5.2 Hypothesis 2: Predicting Clinical Outcomes

Our second set of analyses investigated the associations of parental BAP and adolescent attachment with clinical outcomes.

*Negative Symptom Severity (SANS)*: The final hierarchical regression model predicting negative symptoms was statistically significant, *F*(7, 26) = 2.91, *p* = 0.022, explaining 43.9% of the variance in SANS scores. The inclusion of PAM‐Anxious and PAM‐Avoidant in the final step provided a significant 19.9% of additional explanatory power (Δ*R* ^2^ = 0.199, *p* = 0.008). In this final model, two variables emerged as strong, independent predictors of more severe negative symptoms: higher paternal AQ‐R scores (*β* = 0.45, *p* = 0.016) and PAM‐Avoidant (*β* = 0.46, *p* = 0.005).

*Medication Adherence (MARS‐5)*: The model predicting parent‐reported medication adherence was nonsignificant, *F*(5, 28) = 2.09, *p* = 0.097. Although the covariates in the first step significantly predicted adherence (*R* ^2^ = 0.261, *p* = 0.026), primarily driven by DUP, the addition of parental BAP traits in the second step did not add significant explanatory power (Δ*R* ^2^ = 0.011, *p* = 0.828). In the final model, only a longer DUP remained a robust predictor of poorer medication adherence (*β* = −0.56, *p* = 0.005).

### 3.6 Sensitivity and Contextual Analysis

Finally, a sensitivity analysis was conducted by rerunning the SANS regression model using the standard 50‐item AQ total scores. The pattern of results was highly consistent with the primary analysis, with both higher paternal AQ‐50 scores (*β* = 0.39, *p* = 0.050) and PAM‐Avoidant (*β* = 0.46, *p* = 0.010) remaining significant predictors. This confirms that our findings are robust and not dependent on the specific version of the AQ scale.

To contextualize parental BAP levels in the sample, parental AQ‐50 scores were compared with published neurotypical normative data scored using the same Likert‐based method (*M* = 109.16; Stevenson and Hart 2017). Fathers showed significantly higher AQ‐50 scores than the normative reference group (*M* = 115.67, SD = 14.80; *t*(35) = 2.64, *p* = 0.012). Mothers also had numerically higher AQ‐50 scores (*M* = 114.26, SD = 16.50), although this difference did not reach statistical significance (*t*(38) = 1.93, *p* = 0.061).

## 4 Discussion

The primary aim of this study was to examine how parental BAP traits and adolescent attachment contribute to clinical outcomes in adolescents with non‐affective psychotic disorders. Contrary to our initial hypothesis, parental BAP total scores did not directly predict attachment. Instead, paternal BAP traits and avoidant attachment independently predicted negative symptom severity, while medication adherence was most strongly associated with DUP.

To contextualize our sample, we first compared parental BAP traits with published neurotypical norms and observed that fathers had significantly elevated AQ‐50 scores, whereas mothers showed a smaller, nonsignificant elevation. This pattern may be consistent with recent evidence suggesting that autistic traits are modestly elevated in unaffected first‐degree relatives of individuals with psychosis and may reflect attenuated familial risk expression (Ziermans et al. 2021). Within this framework, the lack of a direct association between parental BAP total scores and adolescent attachment was particularly noteworthy, as insecure attachment is frequently reported in individuals with psychosis (Carr et al. 2018). Theoretical models would predict such a link, given that parental BAP sub‐traits can shape parenting behaviors essential for attachment security. For instance, specific BAP sub‐traits like rigidity and aloofness have been associated with emotion dismissing or reduced positive parenting (DeLucia et al. 2022). This suggests that while a direct link may be elusive, the influence of BAP on attachment likely operates through these more specific pathways.

Our exploratory findings offer additional nuance. Although total BAP scores were unrelated to attachment, lower maternal AQ‐Communication scores—indicating better communication skills—were associated with higher avoidant attachment. This unexpected result may be particularly relevant to our clinical sample. For adolescents with psychosis‐related social processing vulnerabilities, a socially adept or highly engaged parent may inadvertently be experienced as intrusive, leading to defensive withdrawal (Weinreb et al. 2022). This underscores that the influence of parental BAP may be driven by specific sub‐traits rather than a global effect, highlighting the importance of the “goodness‐of‐fit” between parenting styles and the adolescent's social‐emotional capacities. In this context, “goodness‐of‐fit” may refer to how well parental communication and emotional support match the adolescent's social‐cognitive and emotional capacity. A parent who is communicative and engaged may still be perceived as overwhelming if the adolescent has difficulty processing social cues, tolerating closeness, or regulating interpersonal threat. Therefore, the clinical goal may not simply be to increase parental involvement, but to help parents adjust the timing, intensity, and style of support to the adolescent's needs.

A central finding of the study was the significant predictive role of paternal, but not maternal, BAP traits in the severity of negative symptoms. This observation resonates with a growing body of literature suggesting a distinct paternal influence on the expression of neurodevelopmental traits. For instance, fathers often exhibit BAP characteristics more frequently or overtly than mothers, and paternal BAP traits have been shown to have a stronger association with autistic symptom severity in their children with ASD (Di Mento et al. 2024; Narzisi et al. 2025). Our findings extend this concept to the psychosis spectrum, suggesting that the heritable component of paternal BAP may manifest as more severe negative symptoms in adolescents. This association is theoretically consistent given that negative symptoms are relatively more enduring and heritable features of psychosis compared to positive symptoms (Correll and Schooler 2020).

One other plausible explanatory pathway is that paternal BAP sub‐traits, such as pragmatic communication difficulties, social aloofness, and rigidity, may hinder fathers’ ability to provide flexible and emotionally attuned scaffolding. Over time, this mismatch between paternal interaction style and the adolescent's emerging social‐cognitive needs could contribute to entrenched patterns of social withdrawal, reduced motivation, and blunted affect. Although the current design does not allow for mediation testing, these findings underscore the importance of examining specific paternal behaviors, including emotional availability and responsiveness, as potential pathways linking familial autistic traits to negative symptom severity.

Alongside paternal BAP, avoidant attachment emerged as an equally strong predictor of negative symptoms. This is theoretically coherent, as avoidant attachment involves emotional distancing and discomfort with intimacy, which overlap phenomenologically with core negative symptoms such as affective flattening and asociality. Our results build on prior work reporting associations between insecure attachment and more severe psychotic symptoms (Carr et al. 2018). Further contextualizing this relationship, elevated autistic traits—characterized by similar social‐emotional processing difficulties—have also been linked to poorer clinical outcomes in psychosis (Chisholm et al. 2019; Ziermans et al. 2021). Together, these lines of evidence suggest that developmental vulnerabilities in social‐emotional processing, whether expressed as avoidant attachment or autistic‐like traits, may independently amplify negative symptom expression.

Regarding medication adherence, our models found that a longer DUP was the only robust correlate of poorer adherence. This finding aligns with and extends a broader body of research that has consistently linked a longer DUP to poorer long‐term clinical outcomes. While the direct relationship between DUP and treatment adherence specifically has been less studied, our result supports the notion that delays in accessing adequate treatment may establish patterns of disengagement that persist over time (Golay et al. 2016). While parental BAP did not directly predict adherence, the positive association between paternal BAP and DUP hints at a potential indirect pathway. Parental communication or recognition difficulties may delay help‐seeking, thereby extending DUP and indirectly influencing later adherence. Although this potential indirect pathway requires confirmation in future longitudinal research with larger samples, it points to a critical area for early intervention, as suggested by Shalev et al. (2020).

Our exploratory analyses also revealed a significant sex difference, with female adolescents reporting higher anxious attachment. Although this pattern has not been widely studied in psychosis, it is consistent with broader developmental literature showing greater relational anxiety and internalizing tendencies among adolescent females (Falgares et al. 2024; Santona et al. 2022). In psychosis, this may represent an additional point of vulnerability warranting sex‐informed clinical attention.

The present study has several notable strengths. To our knowledge, this is among the first to systematically assess parental BAP in parents of adolescents with non‐affective psychotic disorders and to explore its linkage with adolescent attachment within this specific clinical population. The methodological rigor of the study is enhanced by a homogenous and carefully characterized sample. Specifically, our study focused on adolescents with core non‐affective psychotic disorders (schizophrenia and schizoaffective disorder), while excluding those with more transient or etiologically distinct conditions (e.g., brief psychotic episodes and psychosis due to a medical condition) or intellectual disabilities. Furthermore, all clinical symptom ratings were conducted by a single, blinded clinician using gold‐standard instruments (SAPS/SANS), minimizing potential rater bias. Finally, the robustness of our primary findings was formally tested and confirmed through a sensitivity analysis, strengthening confidence in our conclusions.

### 4.1 Limitations and Future Directions

Several limitations of the present study should be considered when interpreting the findings. First, and most importantly, the cross‐sectional design precludes any conclusions about causality or the temporal nature of the observed relationships. Second, our reliance on self‐report questionnaires for key constructs such as attachment and parental BAP, while common, is susceptible to reporting biases. This is further complicated by the fact that data on parental BAP and medication adherence were collected from the same source, raising the possibility of common method variance. Future studies should incorporate observational or interview‐based measures to provide a multi‐method assessment, including objective adherence indices to mitigate shared‐informant bias.

Furthermore, the relatively modest sample size may limit the generalizability of our results and reduce the statistical power to detect more subtle effects. Although multicollinearity diagnostics were acceptable, the ratio of predictors to observations also raises the possibility of overfitting in the regression models. For this reason, the current findings should be regarded as preliminary and in need of replication in larger, independent samples. Our predominantly inpatient sample may also limit applicability to less severely affected outpatient populations. Longitudinal research with larger, more diverse samples is needed to clarify developmental trajectories and to test whether specific parenting styles mediate associations between parental BAP and adolescent attachment. Finally, the absence of correction for multiple testing may increase Type I error; preregistered hypotheses or statistical corrections should be considered in future work.

## 5 Conclusion

Despite these limitations, our findings have several important clinical implications for the assessment and treatment of adolescents with non‐affective psychotic disorders. The strong influence of paternal BAP on negative symptom severity highlights the necessity of a more inclusive family assessment that extends beyond patient‐focused frameworks. Psychoeducational and supportive interventions tailored for fathers with subclinical autistic traits may enhance family‐based care. Additionally, the robust association between avoidant attachment and negative symptoms suggests that interventions aimed at strengthening attachment security may be an underutilized therapeutic target.

Taken together, this study underscores the importance of a nuanced, family‐systems approach to adolescent psychosis. By moving beyond a singular focus on the patient's symptoms and instead identifying specific parental traits and relational dynamics that contribute to clinical severity, our work provides a rationale for developing more personalized and developmentally informed interventions for this vulnerable population.

## Author Contributions

**Ceren Sena Akyuz Coskuner**: data curation, resources, investigation. **Gokcen Ilcioglu Ekici**: conceptualization, methodology, data curation, formal analysis, investigation, resources, writing – original draft, writing – review and editing. **Esra Cop**: conceptualization, resources, supervision. **Merve Onat**: methodology, data curation, writing – review and editing.

## Funding

The authors have nothing to report.

## Ethics Statement

All procedures were conducted in accordance with the Declaration of Helsinki and received approval from the Ankara Bilkent City Hospital Clinical Research Ethics Committee (No.: E2‐23‐4770).

## Consent

Prior to enrollment, both adolescents and their parents received detailed written and verbal information regarding the study's aims and procedures. Written informed consent was obtained from each adolescent and their respective parent.

## Conflicts of Interest

The authors declare no conflicts of interest.

## Data Availability

The data that support the findings of this study are available on request from the corresponding author. The data are not publicly available due to privacy and ethical restrictions.
